# A Rapid, Antigen-Independent Diagnostic Strategy for Chronic Chagas Disease Based on Serum ATR-FTIR Spectroscopy and Machine Learning

**DOI:** 10.1021/acsomega.6c01730

**Published:** 2026-05-29

**Authors:** Ana Maranni, Thiago Franca, Caique Porsch, Bruno Marangoni, Maria Maranni, Eros de Almeira, Luiz Martins, Paula Andrade, Juliana Ferreira, Solange Domingos, Glaucia Marcon, Cicero Cena

**Affiliations:** † 54534 UFMS − Universidade Federal de Mato Grosso do Sul, Campo Grande, 79070-900, Brasil; ‡ 28132 UNICAMP − Universidade Estadual de Campinas, Campinas, 13083-970, Brazil; § 424846 LACEN- Laboratório Central de Saúde Pública do Estado de Mato Grosso do Sul, Campo Grande, 79080-320, Brazil; ∥ 736264 FIOCRUZ - Oswaldo Cruz Foundation, Campo Grande, 79081-746, Brazil

## Abstract

Chagas disease remains a major neglected tropical disease, particularly during the chronic phase, where diagnosis is challenged by low parasitemia and the extensive genetic and antigenic diversity of *Trypanosoma cruzi*. Current serological approaches require multiple antigen-dependent assays, increasing cost, complexity, and turnaround time, especially in resource-limited settings. Here, we report a label-free diagnostic strategy combining attenuated total reflectance Fourier-transform infrared (ATR-FTIR) spectroscopy of human serum with machine learning to discriminate individuals with chronic Chagas disease from noninfected controls. Serum samples (*n* = 68; 34 positive and 34 negative, previously confirmed by serology and PCR) were analyzed in the 1800–900 cm^–1^ spectral range. Spectral data were denoised using Savitzky–Golay smoothing and fast Fourier transform filtering, normalized by a modified standard normal variate method, and reduced by principal component analysis (PCA). Support vector machine (SVM) classifiers trained on PCA scores achieved optimal performance using a quadratic kernel (*C* = 1). External validation using an independent test set yielded 100% sensitivity, 91% specificity, and 95.5% accuracy, with confidence intervals assessed by Wilson score statistics. Spectral loadings indicate that protein-related vibrational modes, particularly amide I and II bands, dominate class separation, consistent with alterations in humoral immune components during chronic infection. These results demonstrate that ATR-FTIR spectroscopy coupled with machine learning provides a rapid, scalable, and antigen-independent diagnostic approach for chronic Chagas disease. This methodology holds strong potential for point-of-care screening and large-scale epidemiological surveillance, particularly in settings where conventional laboratory infrastructure is limited.

## Graphical abstract

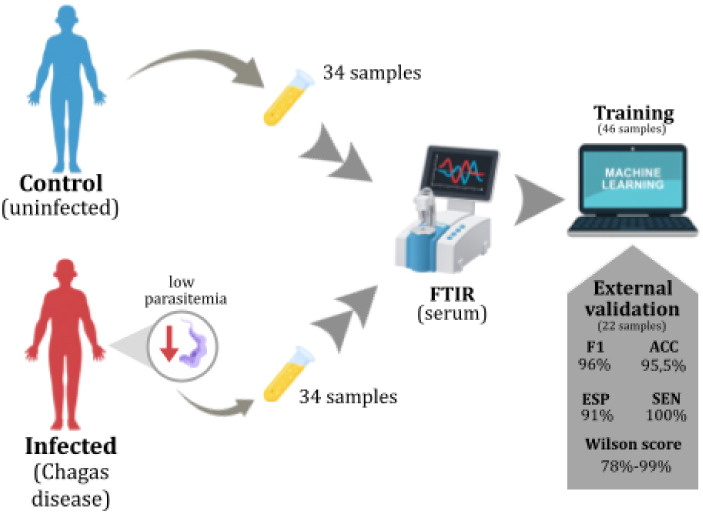

## Introduction

Chagas disease belongs to the group of neglected tropical diseases recognized by the World Health Organization (WHO) and predominantly affects Central and South America, regions considered endemic. Approximately 6–7 million people worldwide are infected with the protozoan *Trypanosoma cruzi* (*T. cruzi*), the etiological agent of Chagas disease, with populations living under conditions of social vulnerability being the most affected.^1,2^ This scenario is largely associated with precarious housing, food insecurity, and inadequate sanitation, which favor infection through contact with triatomine insect vectors and their excreta.

In South America, significant progress has been achieved following the establishment of surveillance networks in endemic regions. Uruguay (1997), Chile (1999), and Brazil (2006) were the first countries to receive certification from the Pan American Health Organization (PAHO/WHO) for the interruption of vectorial transmission of Chagas disease by Triatoma infestans. These achievements resulted from public policies focused on vector control, housing improvement, and insecticide application. Nevertheless, alternative transmission routes, particularly oral transmission and vertical (congenital) transmission, remain prevalent in these countries.^3^ In addition, increased migration to nonendemic regions and ongoing climate change have expanded the geographic risk of infection. As a result, nonendemic countries such as the United States, Canada, Japan, Australia, and several European nations currently report annual cases acquired through congenital transmission, blood transfusion, or organ transplantation.^4^

During the acute phase, most infections are asymptomatic, and diagnosis relies on the high parasitemia characteristic of this stage, typically achieved through direct parasitological detection of the parasite in blood samples. After several weeks, untreated individuals progress to the chronic phase, which may manifest as indeterminate, cardiac, digestive, or mixed (cardiac and digestive) forms. The chronic phase is characterized by low or undetectable parasitemia, making direct detection unreliable. Consequently, diagnosis relies primarily on conventional serological methods, including indirect immunofluorescence (IIF), indirect hemagglutination assay (IHA), and enzyme-linked immunosorbent assay (ELISA). Molecular methods, such as polymerase chain reaction (PCR), which target parasite DNA, offer higher sensitivity and are often used to resolve discordant or inconclusive serological results.^5–7^

Immune responses during both phases of Chagas disease have been extensively investigated. However, studies addressing the acute phase are largely based on experimental models, which limits their direct extrapolation to human disease. In contrast, investigations of the chronic phase are essential for diagnostic purposes, as they are grounded in the host’s cellular and humoral immune responses. Chronic infection is marked by an intensified cellular immune response, particularly reflected by increased frequencies of T lymphocytes (CD4^+^ and CD8^+^ subsets). The activation and maturation of innate and adaptive immune cells are mediated by cytokines, which are present at elevated levels during this stage. These signaling molecules play a crucial role in shaping the humoral immune response, promoting the production of specialized antibodies, predominantly immunoglobulin G (IgG). Among the most representative cytokines are interleukin-10 (IL-10), interleukin-5 (IL-5), interferon-gamma (IFN-γ), and tumor necrosis factor-alpha (TNF-α), reflecting the coexistence of pro- and anti-inflammatory responses. In addition to host-derived immune mediators, parasite-derived proteins also influence the immune response, including surface mucins and antigens released following parasite lysis.^8,9^

Within this context, molecular optical spectroscopy techniques offer a valuable approach for investigating biofluids such as blood serum, which is predominantly composed of immune-response-related biomolecules. Due to its high sensitivity to sample composition, Fourier-transform infrared (FTIR) spectroscopy has been increasingly combined with machine learning (ML) algorithms for the investigation of infectious diseases. Numerous studies have reported diagnostic accuracies exceeding 90% using FTIR-based approaches, establishing robust analytical protocols for continued development in this field.^10–14^ Compared with conventional serological assays, FTIR spectroscopy offers several advantages, including minimal sample preparation, straightforward implementation, and the potential for rapid and high-throughput testing.^15^

A previous study conducted in Brazil by Santos et al. (2025) investigated chronic Chagas disease using FTIR spectroscopy combined with machine learning applied to both liquid and dried serum samples.^16^ For dried samples, the best performance was achieved using logistic regression, yielding sensitivity and specificity values of 93%, whereas for liquid samples, the XGBoost model achieved sensitivity and specificity of 87%.^16^ Although these results are comparable to those obtained with conventional diagnostic methods, the analytical framework relied on multiple modeling strategies with relatively high algorithmic complexity, which may limit interpretability and generalization. In the same national context, serological assays have demonstrated variable performance: ELISA tests showed sensitivities ranging from 92.9% to 100% and specificities from 78.2% to 90%; IIF assays exhibited sensitivities between 87.4% and 100% and specificities between 62.1% and 88.6%; IHA tests showed sensitivities of 71.2% and 93.7% and specificities of 94.9% and 70.9%; rapid immunochromatographic tests achieved sensitivities between 92.8% and 100% and specificities between 78.5% and 92.4%.^17,18^ In this context, the present study advances beyond previous approaches by adopting a more rational and streamlined data analysis strategy, combining dimensionality reduction and a single, well-defined classification model. This simplified yet more robust framework reduces analytical complexity while improving model stability and external validation performance, thereby more clearly revealing the diagnostic potential of FTIR spectroscopy for chronic Chagas disease.

Despite their widespread use, conventional serological assays are hindered by the extensive genetic and antigenic variability of *T. cruzi*, which complicates diagnosis and limits assay performance for geographically distinct parasite strains.^4–9,17–19^ In this context, spectroscopic approaches emerge as a complementary diagnostic strategy, as they align with conventional results while simultaneously providing a global molecular fingerprint of the sample. This feature enables the development of diagnostic protocols that are independent of specific antigens, facilitating disease detection regardless of the infecting parasite strain.

Accordingly, the integration of conventional serological data with computational models derived from spectroscopic information holds substantial scientific and diagnostic value. Such approaches offer opportunities to optimize diagnostic workflows in terms of time and cost, thereby improving healthcare delivery in regions historically characterized by limited access to diagnostic resources. In this study, we explore the combination of ATR-FTIR spectroscopy and machine learning to evaluate its diagnostic potential for chronic Chagas disease using liquid serum samples, which ensure sample compositional homogeneity and facilitate spectral acquisition.

## Methods

### Samples and Data Collection

A data set comprising 68 human blood serum samples was obtained from a collaborative project between the University of Campinas (UNICAMP) and the Oswaldo Cruz Foundation (Fiocruz), following approval by the Institutional Review Board for human research at the Faculty of Medical Sciences, UNICAMP (CAAE: 42742820.7.0000.5404; approval number 6.698.451). Samples were collected from adult residents (>18 years old; mean age: 57 years) of the metropolitan region of Campinas, São Paulo, Brazil, including individuals of both sexes.

According to the Brazilian Clinical Protocol and Therapeutic Guidelines for Chagas Disease issued by the Ministry of Health (PCDT–MS),^20^ samples were considered positive when at least two serological assays based on different analytical principles yielded concordant reactive results. All 68 samples analyzed by the ATR-FTIR–machine learning protocol were previously characterized at the originating center (UNICAMP) using conventional serological tests and PCR, with diagnostic confirmation by electrochemiluminescence (ECL) performed at the Central Public Health Laboratory (LACEN) of Campo Grande, Mato Grosso do Sul. Based on these criteria, 34 samples were classified as Chagas disease–positive and 34 as negative (control group). It is noted that negative classification for Chagas disease does not exclude the presence of other diseases or inflammatory conditions.

ATR-FTIR spectra were acquired from liquid serum samples by depositing 20 μL directly onto the attenuated total reflectance (ATR) accessory of an FTIR spectrometer (Agilent Cary 360). Spectral acquisition was performed over the 1800–900 cm^–1^ range, corresponding to the fingerprint region of biological samples, which contains the most diagnostically relevant vibrational information associated with proteins, lipids, nucleic acids, and carbohydrates. This region is widely used in FTIR-based biomedical studies due to its high sensitivity to biochemical composition and its reduced interference from water absorption compared to higher wavenumber regions.^10–14^ Spectra were recorded with a resolution of 4 cm^–1^ and 64 coadded scans, parameters selected based on preliminary optimization experiments conducted on the instrument.^21^ A water background spectrum was collected prior to each measurement to minimize contributions from H_2_O absorption.

### Data Preprocessing and Model Construction

All data preprocessing and model development were performed using the Scikit-learn library (version 1.4.2) implemented in Python (version 3.12.4).^22^ Spectral preprocessing was initiated by noise reduction. Local low-amplitude experimental noise was attenuated using Savitzky–Golay (SG) smoothing with an 11-point moving window and third-degree polynomial fitting. As high-frequency oscillations persisted, fast Fourier transform (FFT) filtering was applied to suppress interference-related noise components.^23^

Subsequently, spectra were normalized using a modified standard normal variate (SNV) method, which centers the data to zero mean and unit variance while also performing linear baseline correction. Baseline correction was referenced to the transmittance region around 1800 cm^–1^, where no analytically relevant absorption bands were detected.^24^

Dimensionality reduction was then carried out using principal component analysis (PCA),^25^ generating a set of orthogonal principal components (PCs) that preserve the maximum variance of the original data. PCA results were analyzed through score plots, which describe the spatial distribution of samples in the reduced multivariate space, and loading plots, which indicate the contribution of individual wavenumbers to each PC. Outlier detection was performed by evaluating the distance of samples from the multivariate center using Hotelling’s T^2^ statistic.^26^

The observed group separation in PCA space indicated the feasibility of constructing predictive classification models. Accordingly, 70% of the data set was allocated to model training using a support vector machine (SVM) classifier. SVM models were built in the reduced PCA space, with the number of retained PCs selected based on cumulative explained variance. Different kernel functions (linear, quadratic, and cubic) were evaluated, and the regularization parameter C was varied across three levels (1, 10, and 100) to balance margin maximization and model complexity.^27^

### Model Training, Validation Strategy, and Performance Metrics

Model training employed leave-one-out cross-validation (LOOCV), in which one sample is iteratively withheld from the training set and used for validation, while the remaining samples are used for model fitting. This process is repeated until each sample has served once as the validation instance, providing a robust estimate of model performance under limited sample size conditions.^28^ Overall training accuracy was calculated as the mean performance across all LOOCV iterations and used to select the optimal SVM configuration, prioritizing models with lower polynomial degree when equivalent performance was achieved.

To assess generalization performance, the selected SVM model was further evaluated using a hold-out test set comprising 30% of the samples. This subset was randomly separated prior to model training and was not used during any stage of model fitting, hyperparameter optimization, or cross-validation. Although derived from the same overall data set, this set was treated as an independent test set to evaluate model performance on previously unseen dataClassification outcomes were summarized using confusion matrices, from which accuracy, sensitivity, specificity, and F1-score were calculated. In addition, probabilistic confidence intervals were estimated using the Wilson score method, which is particularly appropriate for binomial outcomes and limited sample sizes.

The complete methodological workflow, from spectral acquisition to model validation, is summarized schematically in Figure 1.

**Figure 1:**
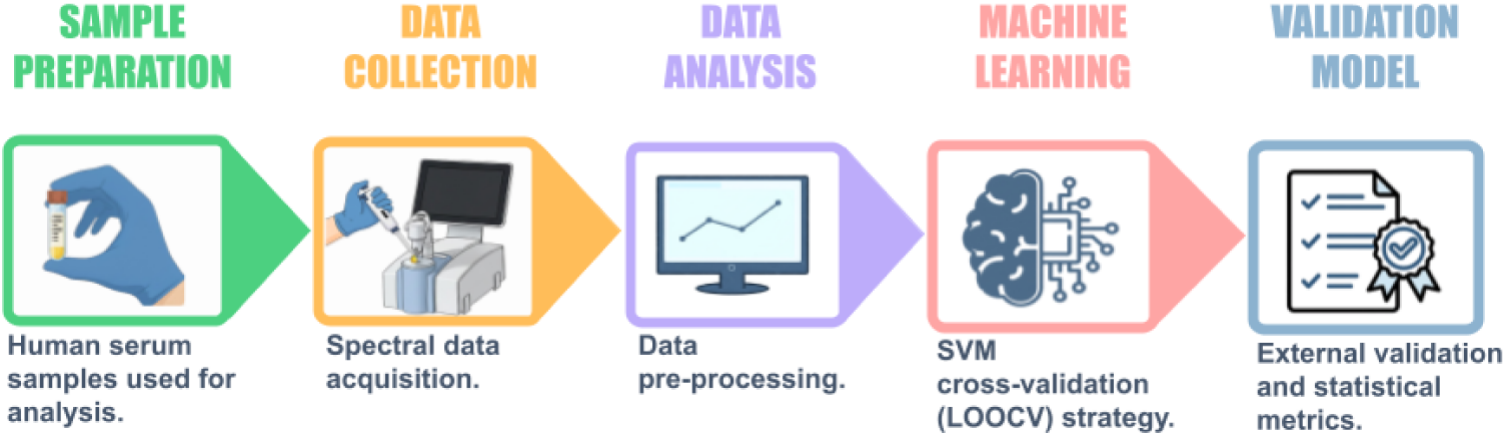
Flowchart of the experimental procedure summarizing the main steps and activities leading to the development of a classification model for the identification of individuals with Chagas disease and noninfected control subjects. Figure created with assistance from AI-generated graphics (Napkin AI).

## Results and Discussion

Figure 2 shows the average SNV-ATRFTIR spectra of blood serum samples from patients affected by Chagas disease, Figure 2a (red trace), and from the control group, Figure 2b (blue trace). In both cases, high-frequency oscillations can be observed, arising from thin-film interference effects caused by the superposition of internal reflection signals at the interfaces between the ATR crystal, the liquid sample, and air. These oscillations become more pronounced due to the reduced signal intensity obtained when analyzing low-concentration liquid samples, since the generated signal is proportional to the amount of material directly interacting with the ATR crystal. In addition, the curvature of the liquid droplet formed upon deposition of the sample on the ATR crystal introduces variations in the optical path length, further enhancing the interference effects.^29^ This phenomenon is accentuated by the inherently low signal associated with the limited concentration of biological material in serum; as only a restricted number of biomolecular components interacts with the infrared radiation, the resulting spectral bands tend to be weak. Moreover, the use of a water background measurement partially removes strong absorption features, further reducing the overall spectral intensity.

**Figure 2:**
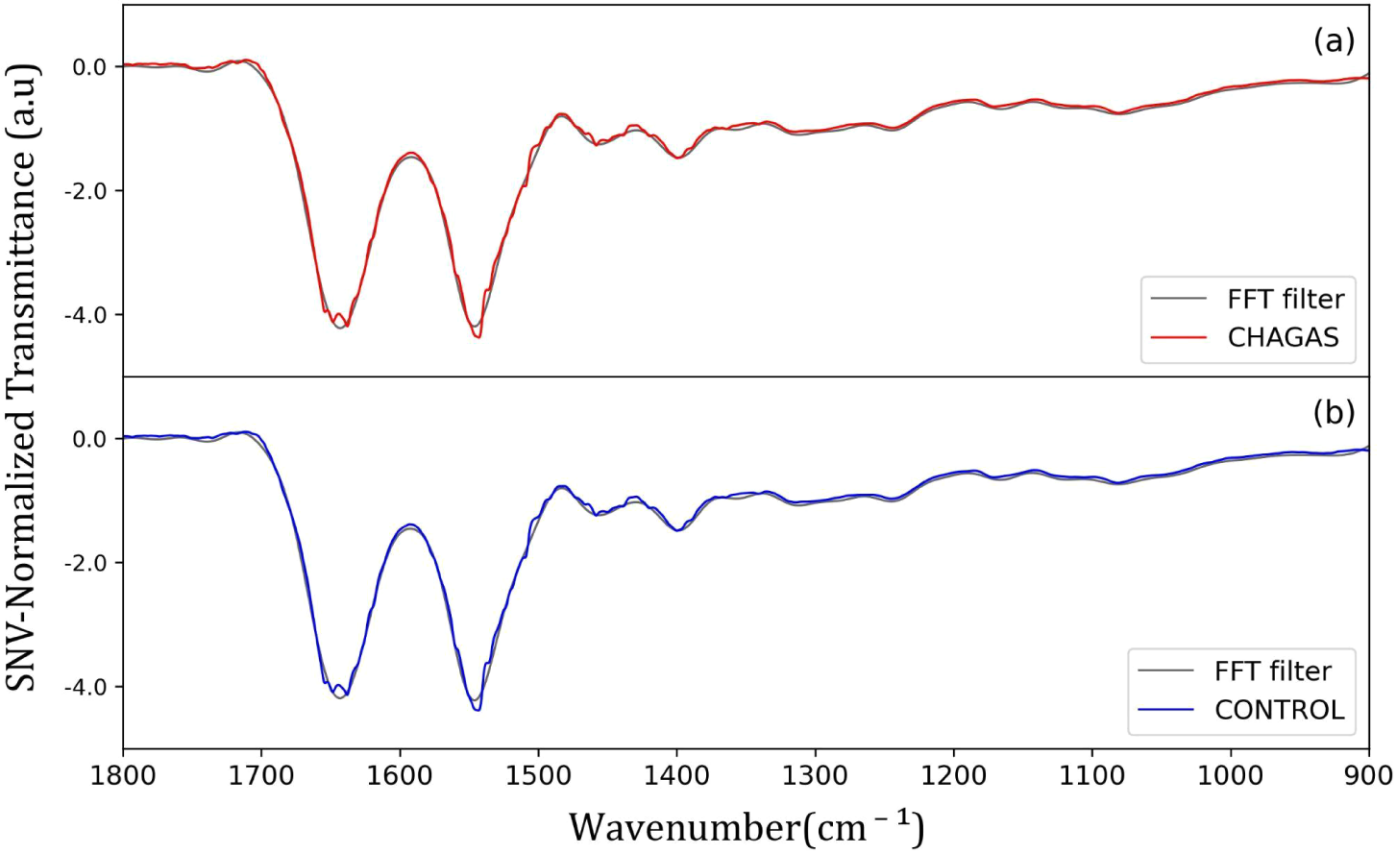
Average SNV-ATR-FTIR spectra of human blood serum samples: (a) Chagas disease-infected individuals (CHAGAS) and (b) control subjects. The colored mean spectra (CHAGAS in red and control in blue) correspond to spectra preprocessed by Savitzky–Golay smoothing and fast Fourier transform (FFT) filtering, followed by modified SNV normalization, and are overlaid on the normalized raw spectra for direct comparison.

In the plots, we also display the normalized spectra after Savitzky–Golay (SG) smoothing and fast Fourier transform (FFT) filtering, shown as gray curves. The SG method effectively corrected low-amplitude local experimental noise through curve fitting; however, it had no significant effect on high-frequency noise components. To address this limitation, FFT filtering was applied, enabling the attenuation of interference-related oscillations and yielding spectra in which the relevant analytical information is predominantly represented by low-frequency signals. The resulting adjustment is close to ideal, as illustrated in Figure 2, since the overall spectral shape is preserved.

All spectra presented were normalized using the modified standard normal variate (SNV) method, including baseline correction referenced to the transmittance region around 1800 cm^–1^, where no analytically relevant absorption features were detected. This preprocessing strategy yielded spectra free from noise-related artifacts and disproportionate amplitude variations. The preprocessing step is essential for multivariate analysis, given the high sensitivity of these methods to data scaling. The use of raw spectra would introduce band intensity as a discriminative factor, leading to inaccurate class differentiation.^30^ For proper modeling, the only relevant discriminative information should be the spectral profile—namely band positions and chemical composition—rather than variability associated with differences in biological material concentration.

The mean spectra presented in Figure 3 indicate that the spectral differences between the classes are subtle, precluding accurate visual discrimination. Nevertheless, compositional analysis of the samples can be performed. In the regions around 1650 cm^–1^ and 1550 cm^–1^, two well-defined bands are observed, which are assigned, respectively, to the C=O stretching vibration characteristic of the Amide I band and to the N–H bending vibration associated with the Amide II band.^30–34^ These vibrational modes are intrinsic to peptide bonds and have previously been associated with the detection of proteins such as immunoglobulin G (IgG).^12^

**Figure 3:**
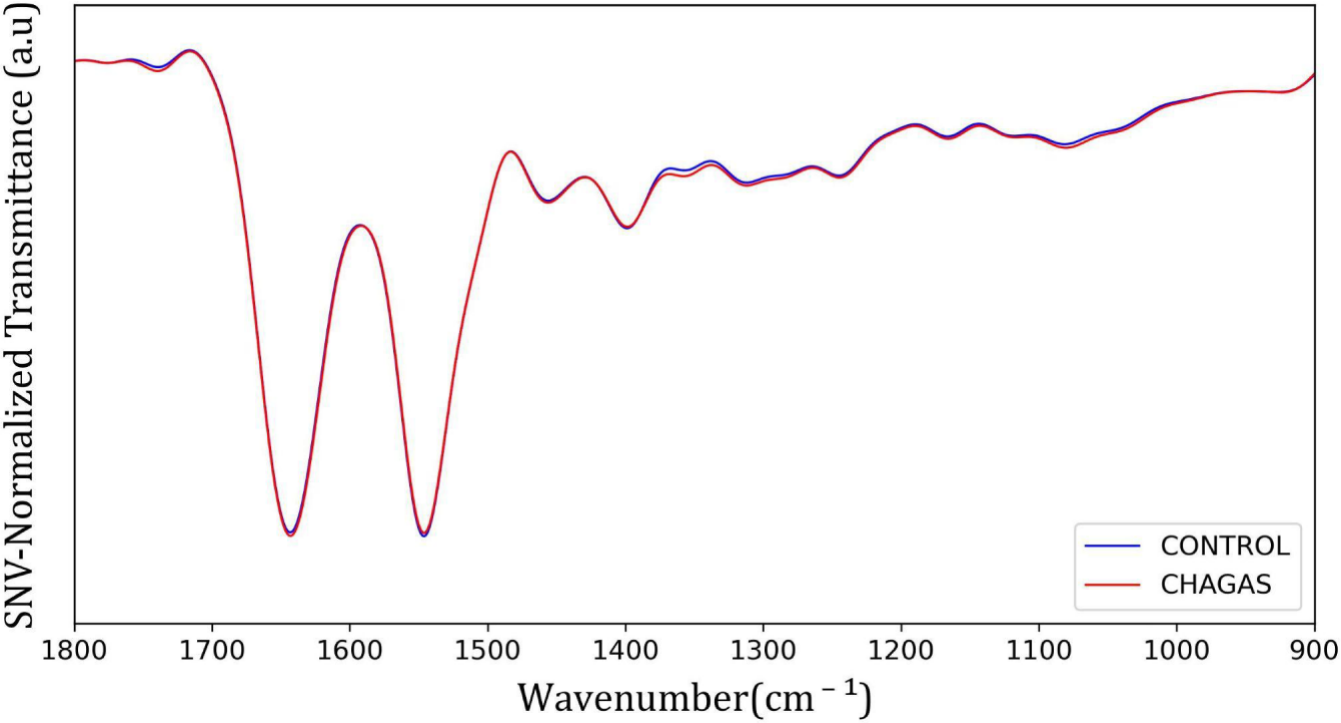
Preprocessed ATR-FTIR mean spectra (SG–FFT–SNV) overlaid for direct comparison of the spectral profiles of the Chagas disease group (red line) and the control group (blue line).

Two additional prominent bands are located around 1460 cm^–1^ and 1400 cm^–1^. The former is attributed to the bending vibration of methylene (CH_2_) groups, predominantly present in lipids, while the latter corresponds to the symmetric stretching vibration of the carboxylate ion (COO^–^), related to carboxylic acid salts that are crucial for maintaining the three-dimensional structure of proteins^12,30–33^

Figure 4 presents the results of the principal component analysis (PCA) performed on the preprocessed spectra. Figure 4a shows the score plot illustrating the spatial distribution of the samples in the plane defined by PC1 and PC2. A clear tendency toward class separation is observed using only these first two components, which retain a substantial proportion of the explained variance: PC1 (59%) and PC2 (21.6%). This behavior indicates the feasibility of constructing predictive models based on support vector machines (SVM). Figure 4b displays the corresponding loading plots, which reveal the contribution of the original variables (wavenumbers) to the formation of the principal components. The gray line denotes the zero-contribution axis; therefore, the observed bands identify the spectral regions that are most relevant for the spatial clustering of samples observed in Figure 4a.

**Figure 4:**
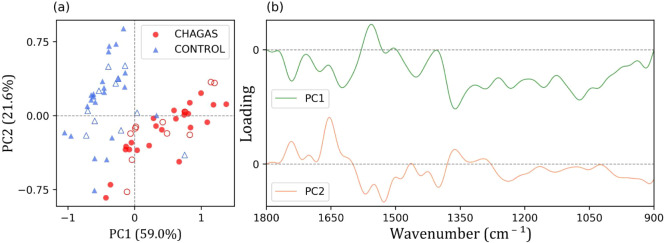
Principal component analysis (PCA): (a) PC1–PC2 score plot showing the distribution of individuals with Chagas disease (red circles) and control subjects (blue triangles). Filled symbols indicate samples used for training by leave-one-out cross-validation (LOOCV), whereas open symbols represent samples used for external validation. (b) Loading plots showing the contribution of each original variable (wavenumber) to PC1 and PC2.

Analysis of Figure 4a shows that the highest sample density is located in the second and fourth quadrants of the score plot. The second quadrant (defined by negative PC1 and positive PC2 values) predominantly clusters control samples. Correlating this region of the score plot with the corresponding loading plots in Figure 4b reveals that the original variables driving the characterization of the control group are those exhibiting (i) negative loadings on PC1, including the region around 1740 cm^–1^ associated with ester C=O stretching vibrations from lipid chains; the 1650–1450 cm^–1^ interval corresponding to Amide I and II bands and lipid contributions; the band at 1360 cm^–1^ attributed to CH_3_ methyl group vibrations abundant in organic compounds; the 1300–1000 cm^–1^ region comprising protein-related bands (Amide III),^31^ ester C–O stretching vibrations, and phosphate group signals from nucleic acids; and finally the region near 920 cm^–1^ associated with C–O–C vibrations of carbohydrates.^12,33^ In addition, (ii) positive loadings on PC2 further emphasize the contribution of amide-related bands in the 1750–1650 cm^–1^ range.

In contrast, the fourth quadrant (defined by positive PC1 and negative PC2 values) contains a substantial proportion of samples from individuals infected with Chagas disease. Examination of the loading plots associated with this clustering indicates that the grouping of infected samples is driven by (i) positive loadings on PC1, particularly the Amide I band around 1650 cm^–1^, and (ii) negative loadings on PC2, again highlighting the 1650–1450 cm^–1^ interval associated with Amide I and II bands and lipid contributions; the region near 1400 cm^–1^ related to protein-associated vibrations; and the fingerprint region between 1300 and 1000 cm^–1^, with dominant contributions from proteins and nucleic acids.^30,32,33^

The combined analysis of score and loading plots demonstrates that proteins play a central role in discriminating between individuals with Chagas disease and control subjects. This finding indicates that components of the humoral immune response, such as cytokines and immunoglobulin G (IgG), act as key determinants for disease identification. In addition, other proteins may contribute to class differentiation, including parasite-derived mucins, which play a protective role for *Trypanosoma cruzi* and are released into the bloodstream following parasite lysis. Despite the evident clustering trend, partial overlap between classes is observed. It is important to note that the control group comprises individuals who tested negative for Chagas disease, which does not exclude the presence of other diseases or inflammatory conditions. Such comorbidities may alter the biochemical serum profile in a manner similar to the infection under study, thereby accounting for the observed dispersion.

Figure 5 presents, in the left column, the score plots illustrating the spatial distribution of samples and, in the right column, the corresponding loading plots indicating the contribution of each wavenumber. Visual inspection allows assessment of the discriminative contribution of each principal component: whereas PC1 (Figure 5a) and PC2 (Figure 5b) exhibit clear class separation, PC3 (Figure 5c) shows substantial overlap between groups, indicating limited discriminatory power. Nevertheless, PC3 was retained for model training to ensure that the cumulative explained variance exceeded 85%, thereby preserving the maximum amount of information from the original variables. Subsequent components were discarded, as they predominantly capture residual variance and instrumental noise without contributing meaningfully to class discrimination.

**Figure 5:**
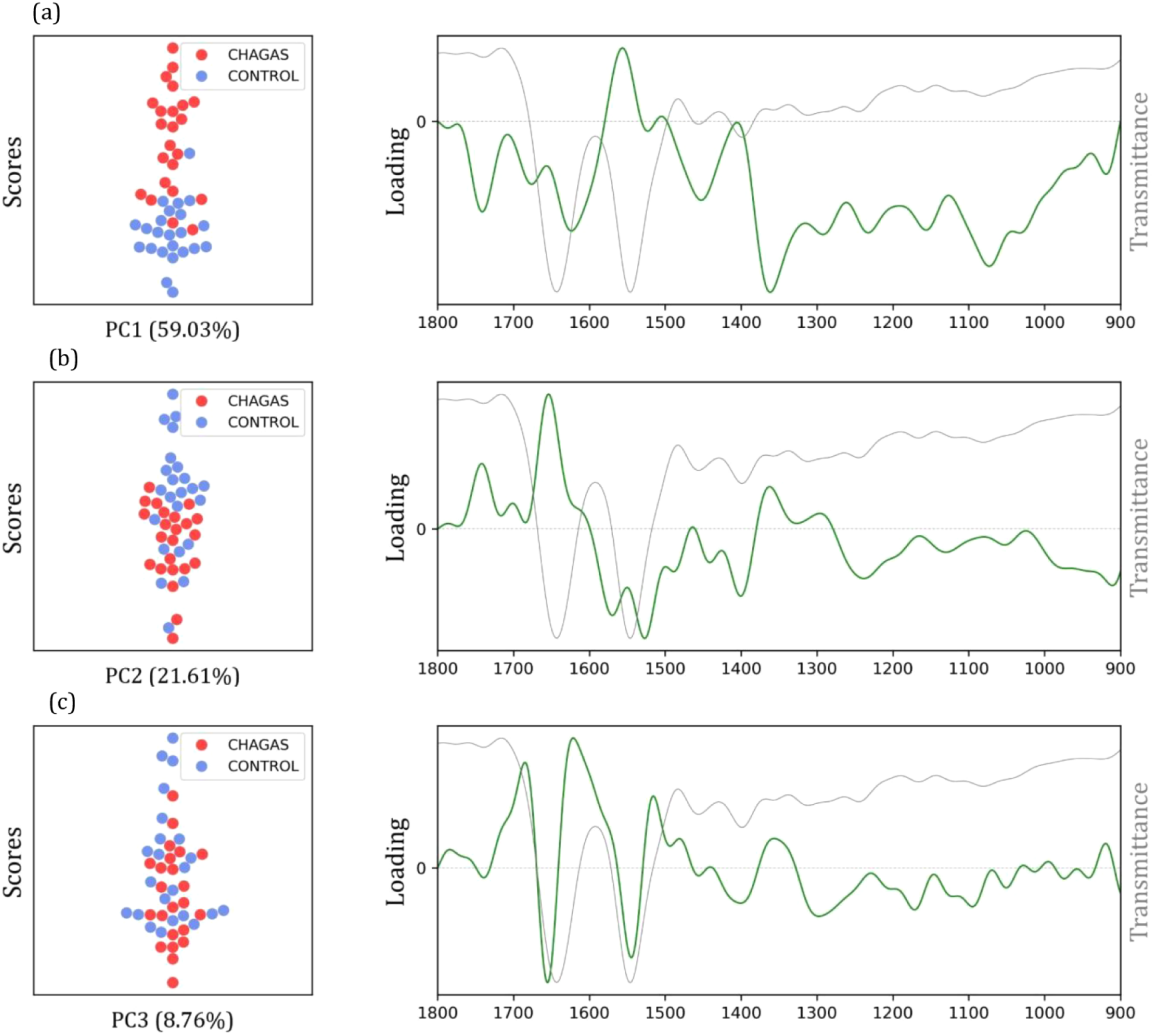
PCA score and loading plots obtained from the preprocessed data of control (noninfected) and Chagas disease groups for (a) PC1 explaining 59.03% of the variance, (b) PC2 explaining 21.61% of the variance, and (c) PC3 explaining 8.76% of the variance. In the background of the loading plots, the mean spectrum of one of the groups is displayed to facilitate peak comparison.

Seventy percent (70%) of the data set was allocated for model training, while the remaining 30% was reserved as an independent external validation set. Model construction was performed in the reduced PCA space using the first three principal components (PCs), which together captured approximately 89% of the total variance. Different kernel functions (linear, quadratic polynomial, and cubic polynomial) and regularization parameter values (*C* = 1, 10, and 100) were systematically evaluated. Hyperparameter selection was guided by performance analysis using leave-one-out cross-validation (LOOCV) applied to the training set, followed by confirmation using the external validation set. The final model was selected based on achieving high accuracy with minimal discrepancy between cross-validation and external validation results, thereby reducing the risk of overfitting or underfitting and ensuring robust generalization.

Figure 6 presents the performance results obtained for the different hyperparameter combinations tested. Identical accuracies were achieved with the quadratic and cubic kernels (*C* = 1) for both the training set and the external validation set. Accordingly, the quadratic kernel was selected over the cubic kernel, prioritizing lower model complexity in the presence of equivalent performance. Increasing model complexity inherently raises the risk of overfitting, which compromises generalization to unseen data.

**Figure 6:**
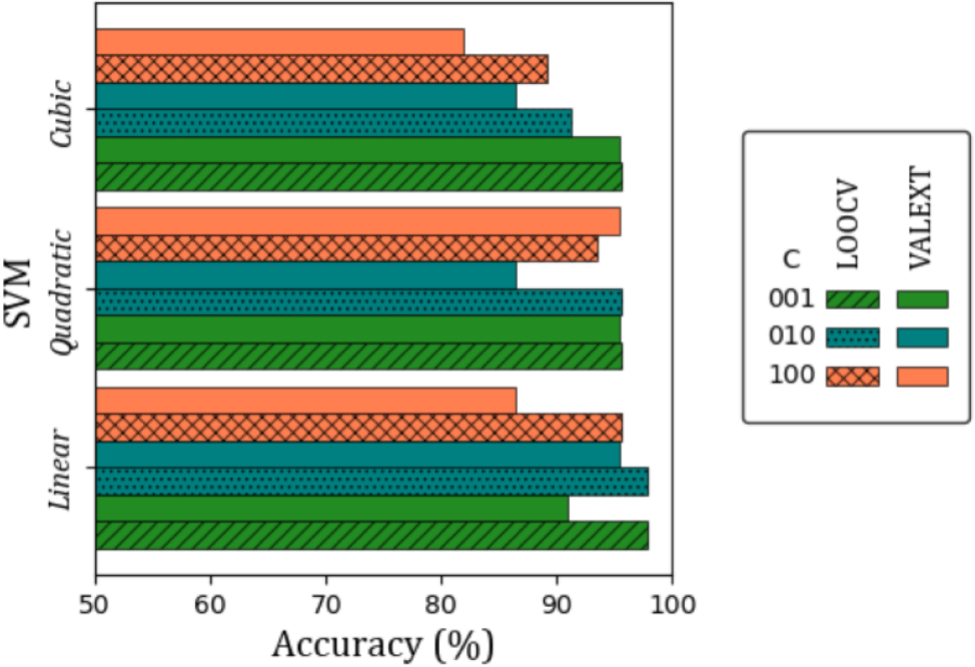
Performance of SVM classification models using linear, quadratic, and cubic kernels (from bottom to top), based on PCA-transformed ATR-FTIR–SG–FFT–SNV spectra from control and Chagas disease groups, evaluated for different regularization parameter values (*C* = 1, 10, and 100).

In contrast, although the linear model achieved excellent accuracy during training, a substantial discrepancy was observed in the external validation results, rendering this kernel unsuitable due to its limited generalization capability.

The classification models exhibited strong performance during the training stage, with accuracies exceeding 80%. For the second- and third-degree polynomial kernels (quadratic and cubic), training accuracy reached approximately 90%. As expected, a reduction in accuracy was observed during external validation, reflecting the application of the models to an independent set of previously unseen samples, for which performance is assessed in a single iteration rather than cumulatively, as occurs during SVM cross-validation.

The results obtained for the quadratic SVM model with *C* = 1, evaluated on a per-sample basis, are presented in the confusion matrices shown in Figure 7. The training accuracy (95.7%), shown on the right side of Figure 7, and the external validation (hold-out test set) accuracy (95.5%), shown on the left side of Figure 7, demonstrate the excellent performance of the selected model. For external validation (hold-out test set), the following statistical metrics were obtained: sensitivity of 100%, specificity of 91%, and an F1-score of 93%. Using the Wilson score method with a 95% confidence level, the resulting confidence interval ranged from 78% to 99%.

**Figure 7:**
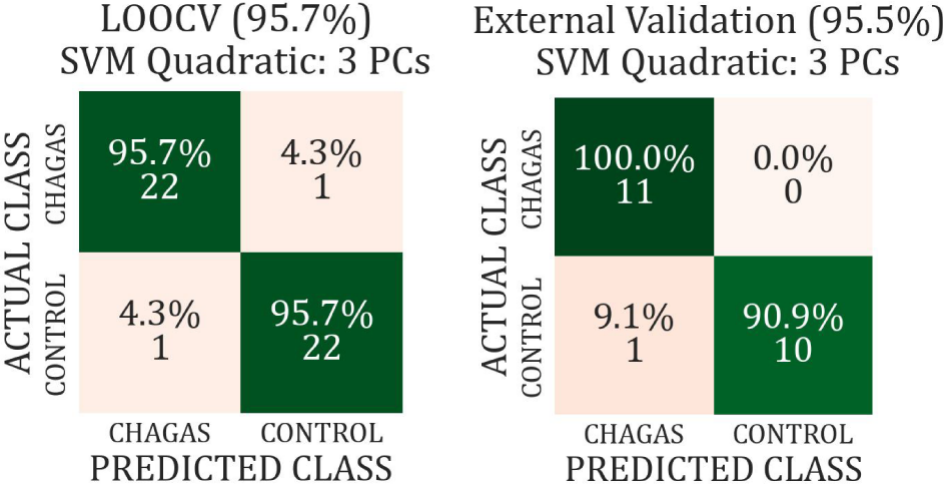
Confusion matrices for the quadratic SVM model (*C* = 1): (a) leave-one-out cross-validation (LOOCV) performed using 70% of the PCA-transformed ATR-FTIR–SG–FFT–SNV data from control and Chagas disease groups, yielding an accuracy of 95.7%; and (b) external validation (hold-out test set) performed using the remaining 30% of the PCA-transformed ATR-FTIR–SG–FFT–SNV data from control and Chagas disease groups, yielding an accuracy of 95.5%.

It should be noted that the external validation performed in this study is based on a hold-out subset derived from the same data set, rather than a fully independent cohort. Therefore, although the results demonstrate strong internal generalization, further validation using larger and independent populations is required to confirm the clinical applicability of the proposed approach.

Investigation of the sample classified as a false positive revealed a relevant observation in its clinical records. In the serological tests performed at UNICAMP, this sample initially yielded a positive result but subsequently tested negative in a follow-up assay (with no conclusive information regarding whether identical methodologies were applied). This discrepancy suggests that the individual may present another disease or inflammatory condition, which could account for the model misclassification. Because ATR-FTIR spectroscopy interrogates the global biochemical composition of the sample rather than a single biomarker, the occurrence of cross-reactivity or spectral overlap with other pathologies is an expected phenomenon.

When compared with previous reports in the literature,^12^ the results obtained in this study indicate superior performance for liquid serum samples. The primary advantage lies in the simplicity of sample preparation and collection, which mitigates the heterogeneity effects frequently observed in dried films,^34^ thereby rendering the method more suitable for large-scale application and facilitating its potential use in field-based diagnostics.

The present work establishes a standardized protocol combining Fourier-transform infrared (FTIR) spectroscopy of blood serum with machine learning for the serological diagnosis of Chagas disease. Nevertheless, further clinical testing is required, including direct comparisons with routinely employed diagnostic techniques and subsequent validation across diverse populations.

Current diagnostic guidelines for Chagas disease require concordant results from at least two distinct serological assays and, in some cases, a third method to resolve inconclusive findings. This multistep workflow results in a time-consuming and labor-intensive diagnostic process. Consequently, there is an urgent need for diagnostic tools that combine rapid execution with high sensitivity, a context in which the methodology proposed herein emerges as a promising alternative.

Conventional serological methods used for diagnosing chronic Chagas disease, such as ELISA and indirect immunofluorescence, involve multiple laboratory steps, with total turnaround times ranging from several hours to an entire day. Molecular techniques, including PCR, although highly sensitive, require DNA extraction, amplification, and downstream analysis, further extending the time to result. In contrast, the ATR-FTIR-based approach enables rapid spectral acquisition with minimal sample preparation and short analysis times, representing a significant operational advantage, particularly for screening strategies and public health applications.

## Conclusion

The results obtained from the ATR-FTIR spectral model, developed to discriminate individuals with Chagas disease from noninfected controls and combined with machine learning, demonstrate strong relevance for diagnostic applications. Using serum samples, the method achieved a sensitivity of 100%, specificity of 91%, and an F1-score of 96%. In addition, Wilson score analysis indicates, with 95% confidence, that the true sensitivity of the method lies within the 78–99% interval. Despite the probabilistic nature of these estimates, independent validation yielded an accuracy of 95.5%, approaching the performance of established reference diagnostic techniques.

The methodology presented here offers a new perspective for rapid, accurate, and low-cost diagnosis of Chagas disease. Within the current diagnostic landscape, this approach represents a valuable complementary tool to conventional methods, such as serological and molecular assays, and may be particularly useful for screening in endemic regions where access to complex laboratory infrastructure is limited. Substantial diagnostic optimization could be achieved through the incorporation of the proposed model into clinical diagnostic protocols for Chagas disease.
